# Fragmented Romanian Sociology: Growth and Structure of the Collaboration Network

**DOI:** 10.1371/journal.pone.0113271

**Published:** 2014-11-19

**Authors:** Marian-Gabriel Hâncean, Matjaž Perc, Lazăr Vlăsceanu

**Affiliations:** 1 Department of Sociology, University of Bucharest, Bucharest, Romania; 2 Faculty of Natural Sciences and Mathematics, University of Maribor, Maribor, Slovenia; University of Zaragoza, Spain

## Abstract

Structural patterns in collaboration networks are essential for understanding how new ideas, research practices, innovation or cooperation circulate and develop within academic communities and between and within university departments. In our research, we explore and investigate the structure of the collaboration network formed by the academics working full-time within all the 17 sociology departments across Romania. We show that the collaboration network is sparse and fragmented, and that it constitutes an environment that does not promote the circulation of new ideas and innovation within the field. Although recent years have witnessed an increase in the productivity of Romanian sociologists, there is still ample room for improvement in terms of the interaction infrastructure that ought to link individuals together so that they could maximize their potentials. We also fail to discern evidence in favor of the Matthew effect governing the growth of the network, which suggests scientific success and productivity are not rewarded. Instead, the structural properties of the collaboration network are partly those of a core-periphery network, where the spread of innovation and change can be explained by structural equivalence rather than by interpersonal influence models. We also provide support for the idea that, within the observed network, collaboration is the product of homophily rather than prestige effects. Further research on the subject based on data from other countries in the region is needed to place our results in a comparative framework, in particular to discern whether the behavior of the Romanian sociologist community is unique or rather common.

## Introduction

De Haan [1] suggests that scientific collaboration could mean different things: co-authorship ties, shared editorship, shared supervision of PhD candidates, common research proposals, co-participation in formal research programs or teams and co-organizing scientific events. There is a consistent literature on investigating the patterns of scientific communities. Research has been approaching different topics, such as citation networks [2]–[4], co-citation networks [5], [6], co-authorship networks [7]–[18], scientometric oriented analysis of authors [19]–[24], mapping idea spaces and science fragmentation [25]–[28], as well as visualization as in Batagelj and Mrvar [29].

We share the view that science, in general, and sociology, in particular, are community-based activities that preponderantly involve dependence and interdependence relationships [30]. For this matter, we aimed at describing and exploring the structure of the co-authorship network identified within Romanian sociology. In doing so, we built on similar studies conducted on co-authorship ties [10], [12], [27], [28], [31], [32]. We expect our research to make contributions to the field and, also, to advance the knowledge on the functioning of the Romanian academic communities.

### Theories of social homogeneity

According to Durkheim [25], social organizations, that lack rules and conventions, lose their ability of coordinating the interdependent units. A fragmented science no longer forms a solidary whole, while field specialization conducts to high levels of isolation among particular groups of researchers. Friedkin [35] suggests that scientific fields are structured as loose and effective networks. Moreover, he indicates that actors’ opinions are affected by the structural configurations of the social positions. In other words, through interpersonal influence mechanisms, actors occupying dominant positions determine the opinions held by the rest of the network.

Investigating how and why people end up in sharing the same ideas, values or practices, put it differently, social homogeneity is one of the research traditions in the field of social network analysis [36]. There are at least two theoretical streams that aim at explaining the sharing of ideas: adapting based models and contagion based models.

*Adapting based models* support the idea that high similarity levels among individuals or other social entities (groups, organizations etc.) are the direct result of isomorphic forces. For instance, formal or legal constraints, the need for legitimacy, occupational socialization or uncertainty are some of the main drivers to force individuals or organizations to mimic the others’ decisions, behaviors, ideas etc [37], [38]. The local information available to individuals is also an important mechanism to explain adapting; especially, when individuals prefer to mimic their friends’ behavior and, thus, deviating from the optimal choices predicted by rational choice theories [39]. The preference of organizations to benchmark their internal processes, activities or behavior as to enhance performance increases the levels of similarity within a specific organizational field [40].

According to the *contagion based models*, individuals influence the beliefs of their social contacts by direct exposure (direct interactions), a mechanism similar to disease transmission [41]. Cliques (maximally connected graphs) allow for a rapid spread of ideas among members. Generally, cliques are composed of strong ties that imply repeated intensive interaction that determine a high level of homogeneity [42]. Social influence can be either interpersonal, or social. In the case of interpersonal or hierarchical influence, a high status individual determines the behavior of the alters [43], [44]. In the case of social influence, cliques force members to adopt certain systems of ideas or practices. Scientists embedded in collaboration networks share ideas, practices and influence each other’s work [27]. The odds for contagion are high in closed triads, where A, B and C share a common identity [45]. Furthermore, social exclusion (forcing the individuals not to adopt specific behavior, ideas, and values) is another mechanism used to explain social contagion [46].

Burt [47] explains social contagion using a cohesion model and a structural equivalence model. The cohesion model is built on social proximity and socialization. When confronted with uncertainty and complex problems, individuals tend to seek advice from friends or colleagues, closed alters. Eventually, the solution is going to split not only over the ego, solving her problem, but also over her closed friends, as it satisfies a common system of values. The structural equivalence model supports the idea that individuals who have structurally equivalent positions tend to influence each other and adopt any ideas or innovations developed by structurally equivalent others and that prove to be successful. Two actors are structurally equivalent if they occupy identical positions or have identical relationships with alters [47]–[51].

To sum up, social network theories of social homogeneity highlight that network configurations determine the distribution of ideas. Describing and exploring the structure of networks could increase our understanding on the behavior of the embedded actors and on their scientific production.

### Collaboration network models in social sciences

The background in social network research on co-authorship structures in sociology and the social sciences is extensive. For example, Moody [27] and Mali et al [30] show that there is empirical support for at least three types of collaboration structures: structures that reproduce the properties of small-world graphs, preferential attachment based structures and structurally cohesive structures.

*Small-world networks (graphs)* are a mix of regular lattices and random graphs, being highly clustered and yet having small path lengths [52], [53]. Collaboration networks that fit small-world models are expected to have many distinct clusters of researchers investigating specific subjects or implementing particular research projects, while the geodesics among these clusters are small (low number of links or degree of separation). This type of structure is especially predicted or expected in cases where researchers and scholars from different fields or disciplines work together in cross-disciplinary projects. As a structural result, for instance, at the level of a specific sector, one might expect to identify densely connected clusters within disciplines and low degrees of separation among disciplines.

Observed collaboration networks can also be modeled as scale-free networks with distributions of degrees that satisfy power-laws. Barabasi and Albert [54] argued that large networks develop under the *principle of preferential attachment*. New links and nodes are not randomly connected to large networks, but based on observed power-law distributions. Basically, all versions of scale-free network structures are based on the model of cumulative advantage in science [30]. This model corresponds to Merton’s [55] observation that science is a reputation market, where rewards are reputation based allocated, irrespective of personal contributions or efforts (“the Matthew Effect”). Moreover, as shown by Moody [27] those entering later are to co-author with “those already in the collaboration network with a probability proportional to their centrality degree”. According to the Matthew Effect, scientific fields grow and develop around highly prestigious scientific scholars.

Moody [27] and Moody and White [56] gave support for *networks defined by structural cohesion*. These networks are densely connected, with no vulnerabilities for fragmentation. In other words, the network remains connected even if specific nodes are to be removed. This type of networks is expected in cases of cross-topic collaboration [30].

Additionally, we mention another type of network structure, the core-periphery model. This model is rather common in the context of social network studies like interlocking directorates [57] or scientific citation networks [58]. Borgatti and Everett [34] formally defined the core-periphery networks as “networks consisting of one group (the core) to which all actors belong to a greater or lesser extent”. As to detect core-periphery structures within social networks, Borgatti and Everett suggested a special algorithm that correlates observed networks with an ideal core-periphery network. High correlation coefficients are considered to be indicative of core-periphery structures within observed relational datasets.

**Figure 1:**
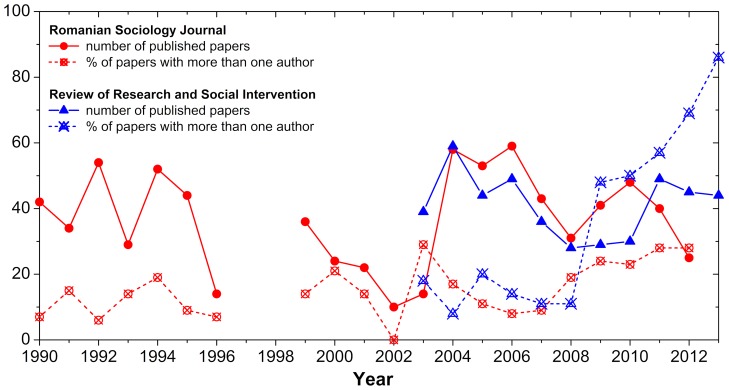
Publishing trends in Romanian sociology. Data is presented for the two main sociology journals that are published in Romania; *Romanian Sociology Journal* (red) and the *Review of Research and Social Intervention* (blue). It can be observed that the fraction of papers with more than one author increases over the years, especially for the *Review of Research and Social Intervention*, while the number of published papers does not display persistent up or down trends. In the years 1997 and 1998 the *Romanian Sociology Journal* went dormant and therefore data are missing.

Glanzel and Schubert [31] or Laband and Tollison [32] reported that the frequency of co-authorships is determined by the specific of each discipline (co-authorships are more common in natural sciences and lesser in social sciences). In our study, we uncovered increasing trends of co-authorships in the Romanian sociology similar to those reported by Hudson [59] and Moody [27]. After inspecting the archives of two Romanian leading journals (*Romanian Sociology Journal* and *Review of Research and Social Intervention* are international database indexed peer-reviewed scientific journals, wherein the great majority of papers is authored or/and co-authored by Romanian sociologists), we have identified consistent growth trends in the percentage of co-authored papers, i.e., papers with more than one author, within the last 23 years, while the number of published papers remains relatively constant (as shown in Figure 1).

The increase in the number of co-authored papers, reported in Figure 1, is indicative of a change in the behavior patterns of Romanian researchers. We argue that investigating the collaboration networks of Romanian sociologists might give us a sense of how sociological research is structurally constructed and of how collaboration is embedded. As a consequence, in this study, we built the co-authorship network of Romanian sociologists and explore its structural characteristics. We consider this investigation to be relevant for understanding to what extent Romanian sociologists share their ideas and collectively influence the research practices within the field.

## Results

### Scientific performance and collaboration

As shown in Table 1, in the field of sociology, Romanian scholars’ papers (irrespective of their being co-authored or not) have a rather low impact. We used *citations,* h-index and g-index scores to measure the scientific impact. As reported, almost half of all scholars have papers without any citations and, consequently, have their h-index and g-index zero. The scientific collaboration within the Romanian academic sociology community (hereafter, ROCOM) is rather weak, with a mean of 1.6 collaborators per scholar and a modal value of zero. Moreover, Romanian scholars are disconnected from the international communities, 85% of them having no foreign collaborators.

**Table 1:** The impact of the Romanian sociology community research productivity.

	Papers	Citations	H Index	G Index	Total collaborators	Foreign collaborators	Romanian collaborators
Mean	10.8	26.8	1.4	2.1	1.6	0.6	1.0
Mode	0	0	0	0	0	0	0
Std. Deviation	17.9	95.1	2.3	3.9	3.0	2.3	1.5
Minimum	0	0	0	0	0	0	0
Maximum	167	1102	17	27	27	24	9
1^st^ Quartile	1	0	0	0	0	0	0
Median	5	1	1	1	1	0	0
3^rd^ Quartile	13	12	2	2	2	0	1

**Note**. N = 267.

### Gender differences

At the level of ROCOM, male scholars, on average, have more years within the higher education system (M = 12.9, SE = 0.78) than female Romanian academics (M = 10, SE = 0.82); the difference being significant t(192) = −2.514, p<.05. Consequently, we discovered a significant association between the sex of the scholars and their academic achievement (i.e. academic title) χ^2^(3) = 20.42, p = .000 (until 2011, in Romania, seniority criterion had an important weight in awarding the academic titles). Furthermore, male scholars have a higher number of publications (M = 12.9, SE = 1.81) than females (M = 8.4, SE = 1.09); the difference being significant t(265) = −2.037. On average, male scholars also have a higher number of citations (M = 37.3, SE = 10.24) than females (M = 14.7, SE = 4.02); the difference being significant t(184) = −2.059, p<.05. When measuring the impact of publications using G index, the gender difference is still significant (t(265) = −2.758, p<.01), with males having, on average, a higher G index (M = 2.75, SE = .37) than females (M = 1.45, SE = .26). However, in terms of co-authored papers (i.e. collaboration degree distributions), we did not find a significant difference between males and females.

### Assessing the fit of different structural models

**Figure 2:**
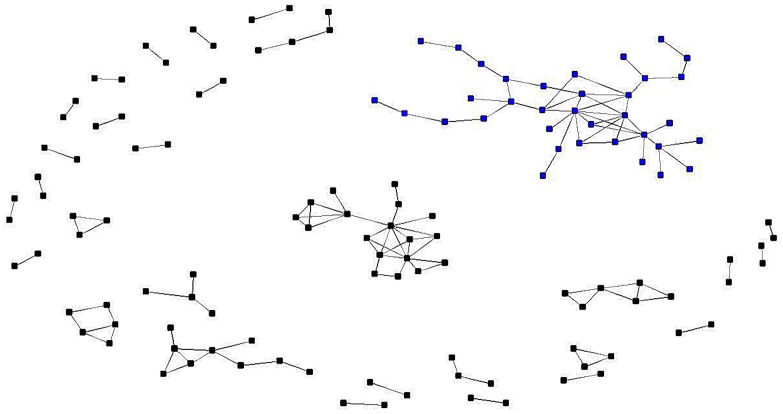
The collaboration network of Romanian sociologists. The largest fully connected component is marked blue. It can be observed that the network is very fragmented, with many pairs, open and closed triplets being completely isolated from each other.

Two researchers have a collaboration tie when they co-author at least one paper. Using this building principle, we generated the collaboration network of all Romanian scholars full time working within all the 17 Sociology Departments across Romania. In Figure 2, one might inspect the collaboration network (the isolates were ignored), which has a density of .004, 264 ties and a high level fragmentation of.976 (the maximum level of fragmentation being 1.0; a pattern wherein all the nodes are isolates). There are also 139 isolates, 19 dyads, three triads, two 4-node components, one 5-node component, one 6-node component, one 9-node component, one 18-node component and a main component of 35 nodes.

We show, in Table 2, the composition of the largest six components contained in the collaboration network. The main component (hereafter, MC) is made up of Romanian researchers whose publishing activity covers 38% of all ROCOM papers and 60% of all citations.

**Table 2:** The composition of the largest six components of the collaboration network of Romanian sociologists.

Components	No. of Nodes	No. of Departments	No. of Papers	% of Papers	No. of Citations	% of Citations	Average H-index	Average G-index	Average years
Main Component	35	7	1088	38	4299	60	4	7	17
Component #2	18	2	294	10	211	3	1	2	8
Component #3	9	1	148	5	590	8	4	5	11
Component #4	6	1	48	2	8	0	1	1	7
Component #5	5	1	38	1	6	0	0.4	1	11

**Note.** As of January 2013, the total number of papers published by the 267 researchers was 2.883, while the total number of citations was 7.167.

**Figure 3:**
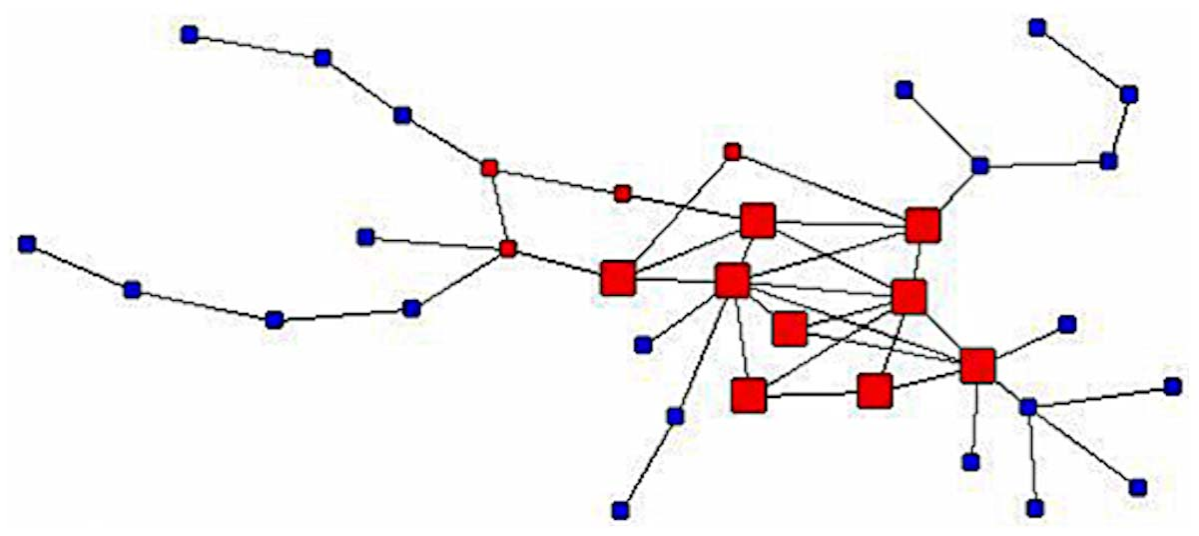
The largest fully connected component of the collaboration network of Romanian sociologists. The largest bi-component is marked red, while the core researchers are denoted using bigger red squares. The later identify the core-periphery structure, as detected within the main component.

It terms of structural pattern models, we tested the fit of different models (i.e. core-periphery, small-world and Erdos-Renyi random graph), both at the level of the whole network and its MC. Using the algorithm for the detection of core-periphery structures [48], we discovered that the collaboration network’s MC imperfectly fits a core-periphery model. The correlation between the ideal structure (the perfect core-periphery network) and the observed collaboration network’s MC is.45 (the maximum value is 1.0). This statistically significant value is strong, but far from a perfect fit with the ideal. The core-periphery structure of the collaboration network’s main component is shown in Figure 3, where the core researchers are denoted using larger red squares. As shown, within the collaboration network’s main component, the core has 9 sociologists and the periphery has 26 (actually, the rest of the sociology within the main component).

We also assessed the fit of the core-periphery model at the level of the whole network. We observed a smaller score of.32, but still statistically significant (i.e. p<.05), indicating a rather weak fit. Because of the high level of fragmentation observed at the level of the whole network, the test for the small world model fit was performed only on the collaboration network’ MC. We obtained a correlation coefficient of −.02 (p>.05). No statistically significant correlation coefficients were obtained when assessing the fit of an Erdos-Renyi random graph model. In the case of the collaboration network, we observed a correlation coefficient of.01 (p>.05) and in the case of the MC a score of.06 (p>.05). Therefore, we failed to fit either a small-world network model or an Erdos-Renyi random graph model to our data.

### Collaboration patterns

Table 3 shows the tests that we have carried our in order to uncover how the co-authorship ties were patterned both within the collaboration network and the main component. At the level of the whole collaboration network, Moran coefficients indicate that collaboration is patterned by *tenure, papers* and *research impact* (i.e. citations, h-index and g-index scores). In other words, it means that scholars prefer to collaborate with others that have similar tenure and scientific activity. The value of the Moran coefficients is indicative of a medium toward strong effect of homophily. The same idea is supported by the Geary’s coefficients, even if this measure is to a certain degree more sensitive to local differences. In case of *gender* and *academic title,* even if both Moran and Geary coefficients are statistically significant, their values indicate a rather weak effect.

**Table 3:** Several tests for assessing patterns within the collaboration network and its main component.

	Collaboration network		Collaboration network MainComponent	
	Geary	Moran	Geary	Moran
Academic title	0.7*	0.3**	0.8	0.1
Gender	0.8*	0.2*	0.7*	0.2
Papers	0.2***	0.7***	0.7	0.2*
G-index	0.5***	0.5***	0.5**	0.2
H-index	0.5***	0.7***	0.5*	0.2
Citations	0.5***	0.7***	0.5**	0.2
Tenure	0.3***	0.8***	0.4**	0.3*

**Note.** Moran’s I statistic of autocorrelation ranges from −1.0 (perfect negative autocorrelation) through 0 (no autocorrelation) to +1.0 (perfect positive autocorrelation). Geary’s C statistic of autocorrelation ranges from 0.0 (perfect positive autocorrelation) through +1.0 (no autocorrelation) to +2.0 (perfect negative autocorrelation). According to Hanneman and Riddle [42], Geary’s C statistic is more sensitive to “local” differences, while Moran’s I statistic is more sensitive to how similar or dissimilar is each pair to the overall average (i.e. “global” difference).

We also tested for patterns within the main component. We have found small significant Moran coefficients for *tenure* and *papers,* and medium significant Geary coefficients for *tenure* and *research impact*. It follows that, at least within the main component, researchers prefer to collaborate with others that are in a similar position in terms of tenure.

We also computed E–I index scores, based on 10.000 permutations, to look for *department affiliation* and *gender* homophily effects. In the case of the whole collaboration network, we found an E–I index score of −.758 (p<.05) for department affiliation and of −.197 (p<.05) for gender. Statistically significant negative E–I index scores were also found within the MC: −.348 (p<.05) for department affiliation and −.391 (p<.05) for gender. These results mean that, generally, both within the whole network and the MC, scholars tend to co-author with colleagues from their own departments and males tend to co-author with males (or females tend to co-author with female colleagues).

### Explaining the collaboration tie degree

We performed several node-level regression models as to explain the collaboration tie degrees of scholars (the models were run using 20.000 permutations). We used the *degree of collaboration ties within ROCOM* as a dependent variable (here, by ‘degree’ we mean the number of symmetric ties that a node has within a network). The dependent variable was regressed on several independent vectors (i.e. tenure, papers, citations, gender, professor academic title, associate academic title and prestige).

As shown in Table 4, all models (except for Model 1, 10 and 11) indicate that the number of papers is the only significant predictor for the collaboration tie degrees. In other words, the greater the number of published papers, the bigger the chances for a higher degree. When using *tenure* (i.e. the number of years a scholar has within the system) as the only predictor, its standardized regression coefficient is statistically significant. This could be explained by the fact that tenure is positively associated with scientific productivity (i.e. papers); the correlation coefficient between the two variables being of.9 (a case for collinearity; for this reason we decided to ignore tenure as a predictor in the other models). On the other hand, when using *citations* as an unique predictor, its standardized regression coefficient is statistically significant. Even if there is no collinearity between *papers* and *citations* (a higher number of papers does not necessary imply a high number of citations), we do suggest that *citations* act as a proxy variable for *papers*. We conclude that Model 8 could be considered as a parsimonious model; a model with only one predictor accounts for 26% of the variation within the collaboration tie degree vector.

**Table 4:** Node level regression for collaboration tie degrees.

	*Collaboration tie degrees within Rocom*										
Variable	Model 1	Model 2	Model 3	Model 4	Model 5	Model 6	Model 7	Model 8	Model 9	Model 10	Model 11
LnTenure	.42***	−.25									
LnPapers		.74***	.61***	.61***	.61***	.59***	.60***	.51***	.50***		
LnCitations			−.12	−.13	−.08	−.10	−.15			.34***	.29***
Gender				.02	.03	.10	.01				
Professor					−.12	−.07					
Associate prof.						.11					
Prestige							.06		.03		.10
*R^2^*	.17***	.28***	.27***	.27***	.28***	.29**	.27**	.26***	.26***	.11***	.12***
*Adjusted R^2^*	.17***	.27***	.26***	.26***	.27***	.27**	.26**	.26***	.26***	.11***	.11***

**Note.** Standardized Coefficients reported, *p<.05, **p<.01, ***p<.001. The node level regression models were performed using 20.000 random permutation. The most important academic titles within the Romanian higher education system are those of a ‘Professor’ and ‘Associate Professor’ (in Romanian ‘Conferentiar’). In our regression models, we created dummy variables for both titles. We also created a dummy aggregated variable called ‘Prestige’, where an ego has 1 if Professor or Associate Professor, and 0 otherwise.

## Discussion

The Romanian academic community within the field of sociology has a low impact scientific productivity and it is disconnected from the international scientific communities. Romanian academic sociologists do not co-author very often, in spite of recent emerging trends. For example, 51% of the full-time working academics do not have any Google indexed co-authored paper. Even if we have discovered gender differences (in terms of the years spent within the higher education system and, consequently, in terms of academic achievement, number of papers and citations), the distribution of collaboration tie degree is not determined by the sex of the scholars. The existing co-authorship ties are defining a collaboration pattern that is highly fragmented and sparse, consequently indicating rather irregular activities of co-authorship. This is consistent with the results reported by other similar studies [27].

Our main aim was to explore and investigate the structural nature of the collaboration network using methods of network science [60], [61], as have been used before successfully to study scientific production and consumption in physics [62], [63], the interaction of people in online affiliation networks [64] the discovery of social events through online attention [65] and the identification of influential spreaders in complex networks [66]. Assessing the fit of different pattern models to the observed network and analyzing how the collaboration ties are patterned, we assume, were substantive ways of learning how Romanian sociologists interact, how ideas are shared and how research practices in the field are influenced and adjusted. Of course, performing the same analysis also on other countries in the region would yield even deeper insights, and it would help place our results in a comparative framework. Previous research on international scientific collaboration [67] and cooperation in the European Union [68], as well as on science indicators of countries [69], [70], is in this regard inspirational. Nevertheless, performing a study such as ours even for a single country requires considerable effort and is not easily transferable to other countries, as it requires regional knowledge, a good command of the language, as well as knowledge about the structure of the system in the country. We therefore hope that the presented research will inspire others to conduct similar research for other (probably their native) countries, and hopefully then the accumulated results will converge to a more comprehensive picture concerning the state of sociology research in the European Union and beyond.

We discovered that neither the collaboration network, nor its main component (MC) does not exhibit the characteristics of a small world model or of an Erdos-Renyi random graph (illustrative for the presence of a ‘Matthew Effect’). The collaboration network did not fit the properties of a small world graph because of its lack of connectivity. While in case of its MC, the correlation coefficient was extremely small and not significant. At the same time, the ‘Matthew Effect’ (which corresponds to star production structures) within the collaboration network and its MC was not empirically supported. In other words, the collaboration network did not exhibit the characteristics of a star producing network model, co-authorship relations not being patterned by the reputation held by egos.

When tested against an ideal core-periphery structure, both the network and its MC were found to be an imperfect match. However, we found a strong significant correlation coefficient (.45, p<.05), but far from being a perfect fit, between the MC and the baseline ideal core-periphery model. The collaboration network’s MC looked as a cohesive structure with a core of nine sociologists, densely tied through co-authorship ties, and a periphery of 26 sociologists lesser connected to the core and sparsely inter-connected. Moreover, the correlation between the whole network and the core-periphery model was statistically significant, but smaller compared with the case of the MC (we argue this value was decreased by the high level of fragmentation the network exhibited).

We also investigated how collaboration ties are patterned across the network. Performing several randomization tests of interval autocorrelations (i.e. the computation of Moran and Geary statistics), we uncovered homophily effects across the network. These results suggested that scholars preferred to collaborate with alters that have similar tenure and scientific activity (comparable numbers of papers, citations). Analyzing the E–I index scores, we also observed department affiliation and gender homophily effects, both within the whole network and its MC.

A last set of empirical results was produced after performing several node-level regression analyses. We observed that the scientific productivity (i.e. the number of papers), alone, accounted for 26% of the collaboration tie degree. Interestingly, the prestige of an ego and her academic titles were not statistically significant predictors for the collaboration tie degree. These results supported the idea that collaboration tie degree was determined rather by the research productivity (‘the more you publish, the more collaborators you have’) and not by prestige (‘the more important you are, the more collaborators you have’).

To sum up, we would highlight three important ideas on the Romanian academic collaboration network within the field of sociology. Firstly, the network imperfectly matches a core-periphery model. Secondly, when it happens, collaboration is the product of homophily effects (tenure, research activity, department affiliation, gender) and not of a Matthew effect. Thirdly, the distribution of collaboration tie degree is, to an extent of 26%, accounted by the research productivity (the number of papers).

It is important to notice, when considering the reported results, that we analyzed relational data (i.e. co-authorship ties) collected using Publish or Perish software package. Implicitly, this means that we took into consideration, when building the collaboration network, only publications (books, papers, reviews etc.) indexed by Google. We do realize that, in some cases, there are co-authored publications not indexed by Google and, for this reason, ignored by our analysis. Because there are no previous studies on Romanian co-authorship relations, we were not able to estimate how well our relational data set matches the real number of co-authored papers and co-authorship network. An alternative tedious and troublesome way to control the quality of our relational data set (that allowed for the construction of the collaboration network) is to inspect academics’ vitae. Or else, we could try to analyze how Google works while making its indexing and see what, if any, types of publications are not indexed.

### Conclusions

Summarizing, we conclude that co-authorship in Romanian sociology is more of an exception rather than common practice. Yet, collaboration does exist and it tends to look like a core-periphery model. Data suggest that, on the one hand, there is a relatively small core of academics densely tied up, and on the other hand, there is a comparatively large sparse scattered periphery. This indicates that both the circulation of ideas as well as actual scientific collaboration are significantly restricted. The structure of the interaction network suggests further that the spread of innovation and cooperation within the field is structurally limited. However, if some new ideas or major advances would spill over the entire community, then it is very likely that the mechanism to facilitate this process will not be the direct inter-personal communication. Rather, our research suggests that social contagion, if any, will be conveyed by processes similar to structural equivalence contagion. Put differently, advances within the field of Romanian sociology are to be imported and transferred from a distance.

## Methods

The first step in our study was to seek out all collaboration signals within the Romanian sociology community. The growing trends, reported in Figure 1, were treated as a proxy for any significant co-authorship activities. In the next step, we built the co-authorship network using as a data source the Harzing’s Publish or Perish (PorP) software package [33]. In January 2013, the 17 Romanian sociology departments employed 267 academics in a full-time manner. The publications of these individuals were retrieved and archived with the PorP software package. The co-authored papers were used as the backbone of the collaboration network, whereby two researchers were considered to be connected if they co-authored at least one paper. In the resulting collaboration network, we kept only the co-authorship relations among the 267 sociologists (as our interest was to study how Romanian academics interact).

Furthermore, we have collected several attribute data for all the 267 full-time employed Romanian sociologists, including gender, academic title, number of papers, number of citations, Hirsch’s h-index [71], [72], as well as tenure (i.e. years between the first and the last indexed publication). Additionally, for each of the 267 academics, we computed both the number of all their co-authors within and outside of Romania.

We have also described and explored the structural properties of the collaboration network using several routine procedures available within the UCINET 6.0 environment [73]. The graph visualizations were made using NetDraw 2.0 [74]. Simulations of small-world random networks were performed using Pajek, while Erdos-Renyi random graphs were generated with UCINET 6.0. The assessment of how different structural models fit to our observed relational data was carried out by using the Core-Periphery algorithm [48] and quadratic assignment procedure correlations.

Lastly, we have analyzed the patterns of collaboration within the network of Romanian sociologists by performing randomization tests of interval autocorrelations and E–I index computations. Given a partition of a network into a number of mutually exclusive groups, the E–I index is the number of ties external to the groups minus the number of ties that are internal to the group divided by the total number of ties. A permutation test is performed to test whether the E–I index of a network is significantly higher or lower than expected. We have also performed an in-depth node-level regression analysis in order to explain the degree of collaboration ties.
